# Buccal Bone Changes Around First Permanent Molars and Second Primary Molars after Maxillary Expansion with a Low Compliance Ni–Ti Leaf Spring Expander

**DOI:** 10.3390/ijerph17239104

**Published:** 2020-12-06

**Authors:** Valentina Lanteri, Davide Cavagnetto, Andrea Abate, Eleonora Mainardi, Francesca Gaffuri, Alessandro Ugolini, Cinzia Maspero

**Affiliations:** 1 Department of Biomedical, Surgical and Dental Sciences, School of Dentistry, University of Milan, 20100 Milan, Italy; valentina.lanteri@unimi.it (V.L.); davide.cavagnetto@gmail.com (D.C.); andreabate93@gmail.com (A.A.); elemainardi95@gmail.com (E.M.); francesca.gaffuri@unimi.it (F.G.); 2 Fondazione IRCCS Cà Granda, Ospedale Maggiore Policlinico, 20100 Milan, Italy; 3 Department of Sciences Integrated Surgical and Diagnostic, University of Genova, 16132 Genova, Italy; alexugolini@yahoo.it

**Keywords:** maxillary expansion, 3D imaging, CBCT, Leaf springs expander, bone thickness

## Abstract

*Background:* Vestibular bone thickness changes and dento-alveolar buccal tipping of second primary molars and of first molars after maxillary expansion performed with a slow maxillary expansion protocol was investigated. *Methods:* Twenty patients (mean age 7.3 ± 0.9 years old; 9 male and 11 female) were treated according to the Leaf Expander protocol. Buccal alveolar bone thickness (BT), buccal alveolar bone height (BH), inter-dental angle (TIP), and inter-molar width (IW) regarding first molars and second primary molars were calculated before and after expansion on cone beam computed tomography (CBCT) images. Descriptive statistics and paired t-tests were used to assess changes between the pre-treatment and post-treatment measurements. *Results:* Bone thickness vestibular to second primary molars and intermolar width of both teeth were the only variables that showed statistically significant changes. *Conclusions:* It appears that buccal bone thickness vestibular to first molars was not significantly reduced after maxillary expansion with the Leaf Expander. The clinical use of a slow maxillary expander with Ni–Ti springs appears efficient and safe in in the correction of maxillary hypoplasia during mixed dentition.

## 1. Introduction

Maxillary hypoplasia and its consequent most common epiphenomenon, that is, posterior crossbite, is a quite common finding in orthodontic practice as it is among the most widespread malocclusions in pediatric patients [1,2]. Its prevalence has been reported as between 5% and 22% [3,4]. The most recognized etiological factors for posterior crossbite are the transverse maxillary constriction due to dental, skeletal, or neuromuscular components. This malocclusion may cause functional mandibular shift, and, in time, a skeletal asymmetrical discrepancy [5]. 

Maxillary expansion has been widely used and accepted to correct posterior crossbite and the transverse maxillary deficiency in growing patients, with the goal of opening the mid-palatal suture, providing appropriate and stable transversal width increase avoiding future extraction [6,7,8]. Different types of tooth borne appliances and different expansion protocols such as rapid maxillary expansion (RME), slow maxillary expansion (SME) and semi-rapid maxillary expansion have been tested over the years to achieve the best results in mixed dentition. However, no clear evidence exists between the different appliances nor between the different screw activation protocols on which is best to obtain the maximum orthopedic expansion with the least dental side effects on anchorage teeth [9]. 

Among the devices for palatal expansion, we can operate a major distinction between rigid screw-activated appliances (e.g., Haas, Hyrax, and so forth), whose activation is always characterized by intermittent and high forces, and that can be activated following rapid or slow maxillary expansion protocols depending on the frequency of screw activation [10,11,12,13], and other appliances that have an elastic screw-activated modulus usually made of nickel–titanium (e.g., Leaf Expander, Ni Ti palatal expander and so forth) that provides a slow maxillary expansion with low and constant forces, they require less compliance by patient’s parents and are usually more comfortable for pediatric patients [14,15,16]. 

The available evidence suggests that both types of expanders provide efficient maxillary expansion [17]. Both kinds of expansion cause—while orthopedically expanding the maxilla—a simultaneous buccal bending of the lateral alveolar processes due to the resistance offered by the distracted palatal fibrous mucosa and a buccal displacement of the anchorage teeth that suffers from the dental side effects of a tooth borne maxillary expander [18,19]. Periodontal and bone thickness effects following palatal expansion have been evaluated by a few studies with conventional radiographic examination such as lateral cephalograms, panoramic, and/or periapical radiographs [20,21]. 

Cone-beam computerized tomography (CBCT) has gained an increasing popularity in the last decade because of its numerous advantages while maintaining relatively low doses of radiation exposure [22,23]. A recently published article started to describe the three-dimensional quantitative changes (at the skeletal, dentoalveolar, and periodontal level) following rapid and slow palatal expansion [24]. Both protocols cause different degrees of buccal bone loss, but the results are so confounding/confusing because of the variety of appliances used for the expansion and because of the different measurement methods. Recently, a new type of slow palatal expander with two nickel–titanium leaf springs (Leaf Expander^®^, Leone, Italia) as an active part has been introduced [25]. This appliance is similar to a Hyrax expander, with the only difference in its active part since the Leaf Expander has a Ni–Ti leaf spring that can be activated through a midpalatal screw and delivers lower calibrated and continuous forces to perform palatal expansion. Its main goal is to provide a compliance-free slow maxillary expansion with an optimized force system [25,26]. Depending on the spring chosen, these appliances can deliver a constant lateral force of 450 g or 900 g. It requires no compliance from the patients’ parents, and when compared to conventional expanders, it was demonstrated to be less painful and able to provide a similar amount of expansion [27,28]. To date, the published data appear promising [29,30]. Therefore, the aim of the present observational retrospective study was to evaluate the dento-alveolar buccal tipping and the bone thickness changes around the upper first molars and deciduous primary molars (anchorage teeth) immediately after Leaf Expander treatment in patients with mixed dentition by means of CBCT scans.

## 2. Materials and Methods 

A retrospective observational study was conducted at the Department of Biomedical Surgical and Dental Science, University of Milan, Milan, Italy. All records consisted of CBCT images obtained before and after the maxillary expansion treatment of patients treated between February 2017 and January 2020 at the Department of Biomedical Surgical and Dental Science, Fondazione IRCCS Cà Granda Ospedale Maggiore Policlinico. The patient’s parents or guardians signed an informed consent to allow us to use patient’s records for research purposes. The present research was approved by the Ethical Committee of the Fondazione IRCCS Ca’Granda, Ospedale Maggiore, Milan, Italy (Protocol No. 573/15).

The inclusion criteria of the present study were as follows: children of 6–10 years old; maxillary constriction (intermolar width <30 mm) w/o unilateral posterior crossbite or mandibular shift cases, mixed dentition with E+E well preserved; and no previous orthodontic treatment. The exclusion criteria were the presence of complex malocclusions; deterioration or lack of teeth on which to set the device (E+E); presence of oral or gingival pathologies; patients with documented pathologies (congenital deformities or gained pathologies) of upper airway; patients who had received surgery of the airway space; patients with obstructive sleep apnea syndrome (OSAS); insufficient oral hygiene (full mouth plaque score >30%); and inadequate diagnostic records.

**Figure 1:**
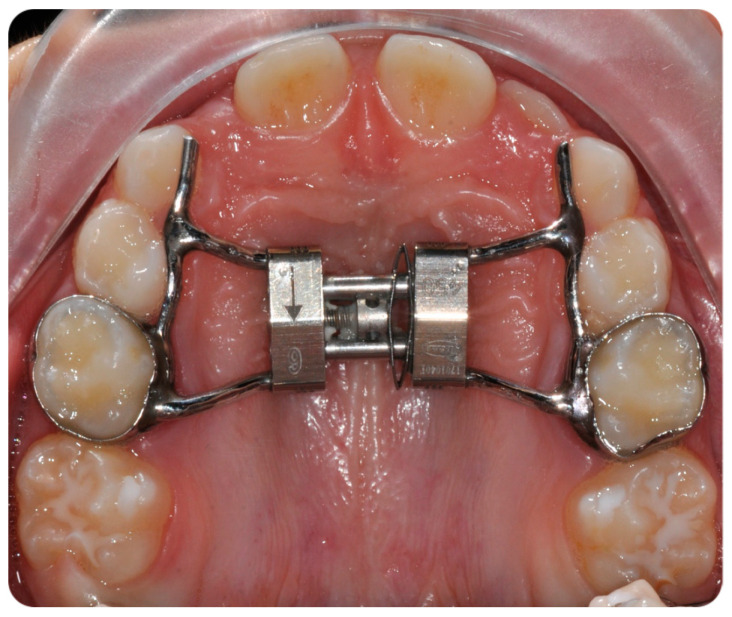
Leaf expander with anterior arms extended up to the deciduous canines.

The final sample included twenty patients (mean age 7.3 ± 0.9 years old; 9 males and 11 females), treated by the same operator (V.L.) with Leaf Expander therapy [26]. All devices were cemented on the primary second molars [31] using a glass-ionomer luting cement (Multi-Cure; Unitek, Monrovia, CA, USA). Patients that underwent maxillary expansion with a Leaf Expander followed a specific protocol designed for this appliance, which consists of a continuous lateral force of 450 g. (Figure 1).

The Ni–Ti spring was blocked using a ligature wire when patients presented dental overcorrection and the expansion was considered achieved. The expansion was considered complete when the occlusal aspect of the maxillary lingual cusp of the first upper molars contacted the occlusal aspect of the vestibular cusp of the mandibular first molars. After an average time of 10.8 months including usually two months of active phase and eight months of retention, the appliances were removed by the clinician.

### 2.1. Cone-Beam Computerized Tomography (CBCT) Examination and Data Processing

All the CBCT scans were acquired with the same device, which was an iCAT ® FLX V-17 series cone-beam dental-imaging system (1910 N. Penn Road, Hatfield, PA 19440, USA). During the acquisition, patients were positioned in the natural head position (Frankfurt plane parallel to the floor). A voxel size of 0.4, a slice thickness of 0.4 mm, and different field of view (FOV) dimension depending on clinical needs were set.

After acquisition, an expert radiologist saved the scans in digital imaging and communications in medicine (DICOM) format, then an orthodontist specialist in 3D imaging analyzed them using Horos 3.0 software (Horos Project, Annapolis, MA, USA).

CBCT images are characterized by high definition and sensitivity of the scans, which guarantees that the cortical bone thicknesses, together with teeth, were visualized without any distortion and overlapping.

**Figure 2:**
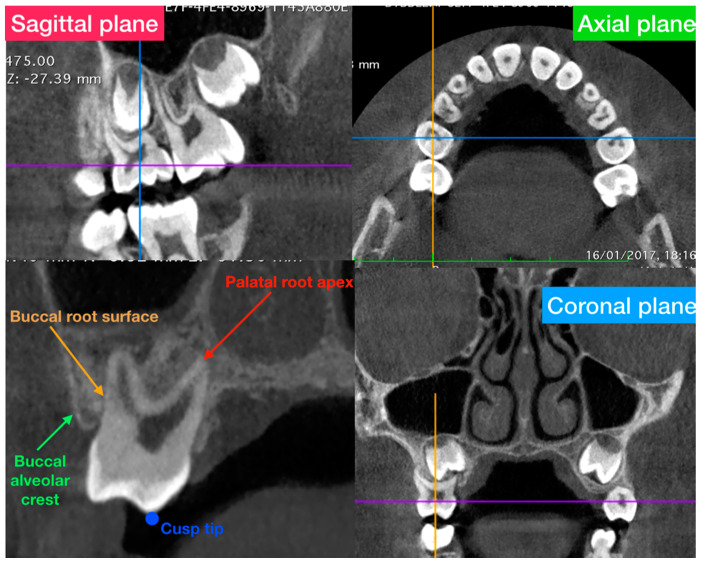
General overview of the multi-planar reconstruction mode of the software and definition of the anatomical structures used in the present study to evaluate the alveolar bone thickness.

The Horos software permitted viewing the CBCT exam in the multiplanar reconstruction mode: three sections that correspond to each plane of space referring to the sagittal, axial, and coronal plane (Figure 2).

Axial-guided navigation (AGN) was used to locate landmarks [31] by moving the axial cursor on the sagittal or coronal multiplane reconstructions guided by the axial plane along the axis of the dental root to achieve an optimal visualization of the crown, root, and marginal alveolar bone in the chosen view.

**Figure 3:**
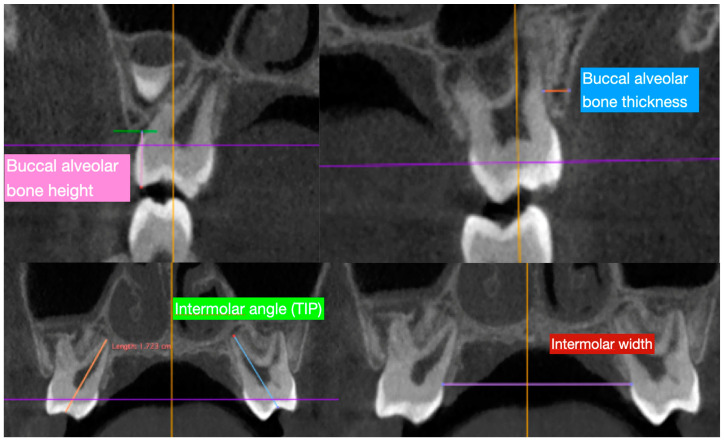
Measurements traced in the present study in order to evaluate the characteristics of the alveolar bone thickness and tipping of the second deciduous molar and first permanent molar. Abbreviations: BT = Buccal alveolar bone thickness; BH = Buccal alveolar bone height; TIP = Interdental angle; IW = Intermolar width.

The parameters measured (Figure 3) before and after maxillary expansion treatment have been previously described by Brunetto et al. [24]:

- Buccal alveolar bone thickness (BT), defined as the distance between the buccal root surface and the outer border of the alveolar bone, along a horizontal line passing through the furcation;
- Buccal alveolar bone height (BH), defined as the distance between the buccal or mesio-buccal cusp tip and the buccal alveolar bone crest;
- Inter-dental angle (TIP), represented by the angle between the right and left axes of the upper molars (6-6) and deciduous molars (E-E), determined by connecting the central fossa and the apex of the palatal root; and
- Inter-molar width (IW), defined as the upper inter-molar (6-6) and deciduous molar (E-E) distance between the mesio-buccal cusp tips.

Buccal alveolar bone thickness and alveolar height were calculated on the right and left sides for upper molar (6 and 6) and deciduous molars (E and E) before and after expansion.

### 2.2. Statistical Analysis

G*Power (version 3.1.9.4, Franz Faul, Universitat Kiel, Kiel, Germany) was used to calculate the sample size using a power (1 − β) of 0.90, an alpha of 0.05, and the mean and standard deviations of the distance between the buccal CEJ and the most occlusal point of the of the buccal alveolar crest (NOV) after RME obtained by Brunetto et al. [24].

The analysis showed that 15 patients were needed to execute a statistically meaningful comparison. All data were carried out by the first observer (E.M.). Fifteen randomized CBCT were selected from the archives of the patients used in the present study and all measurements were re-traced 15 days after by the same examiner and checked by a second observer (A.A.) to evaluate the method error; they were unaware of the patients to which each record belonged. Then, the intra-operator and inter-operator reliability was calculated using the intra-class correlation coefficients (ICC).

The Shapiro–Wilks test found a gaussian distribution of the data. Thus, the parametric test was used in the statistical analysis. Descriptive statistics with mean, standard deviation (S.D), confidence interval, and I and III interquartile were computed at time T1 and T2 for all the variables previously mentioned. The paired t-test was calculated to evaluate significant changes between the pre- and post-treatment measurements. *p* value less than 0.05 was accepted as significant (*p* < 0.05).

## 3. Results

Intra-class correlation coefficient values testify a high agreement of the intra and inter-operator reliability, demonstrating the reproducibility of the method. Correlation coefficient results were larger than 0.96 (Confidence Interval 0.95–0.98). The method error was considered negligible. To analyze the data obtained, descriptive statistics and a statistical comparison were performed. Descriptive statistics are reported in Table 1; means, standard deviations and the confidence interval were assessed for each parameter before (T1) and after (T2) treatment (Table 1). 

Statistically significant differences observed after maxillary expansion for all variables measured are shown in Table 1. The results showed a statistically significant reduction (*p* < 0.01) of the buccal alveolar bone thickness (BT) with a mean difference between pre- and post-treatment of 0.54 mm on the right side and 0.47 on the left side for the second deciduous molar. A statistically significant increase (*p* < 0.05) was noticed in the buccal alveolar bone height (BH) of 0.72 mm on the right side and 0.33 on the left side at the level of the second deciduous molar (Table 1).

The intermolar width of the upper deciduous second molars and the upper first permanent molars became statistically bigger after the expansion protocol (*p* < 0.01). On the other hand, the reduction in BT and the increase in BH for the right and left first permanent molar were not significant as well as the increase of the tipping tooth movement for the second deciduous molars and first permanent molars (Table 1).

## 4. Discussion

The primary endpoint of this work was to evaluate changes in the buccal bone thickness of E teeth and first molars and the dento-alveolar buccal tipping on the same teeth in pre-pubertal patients treated with the Leaf Expander appliance as evaluated by CBCT scans. A progressive expansion was delivered each week by the Ni–Ti spring deactivation. The purpose of the use of constant low forces is twofold, that is, provoking more physiologic responses from the midpalatal suture [32,33] and obtaining 0.5 mm of expansion per week, which, as Proffit et al. [34] stated, is the amount of expansion that the tissues of the mid-palatal suture can tolerate before compensation mechanisms occur.

The changes in the intermolar width of the first upper molars are in line with previous findings of vestibular tipping and displacement following rapid or slow maxillary expansion with conventional rigid framework appliances [35,36]. The present research shows that the increase in intermolar width (MW) at the level of E-E and 6-6 was significant and demonstrates the capability of this low compliance Ni–Ti expander in increasing the transverse diameter of the palate with less discomfort for the patients.

The interdental angle (TIP) was measured to observe the vestibular-inclination of the dental elements and showed excellent results as its increase was not significant either at the level of E-E or of 6-6.

This means that there was primarily bodily movement of the teeth and allows us to state that there was a real expansion of the maxillary transverse diameter without being linked to compensatory tipping movement. Rungcharassaeng et al. [37] reported a greater increase in vestibular tipping (6.64 mm) of the upper fist molars, allegedly due to the differences in type of appliance and in the amount of activation of the screw.

The authors of the present study found a statistically significant decrease in the buccal alveolar bone thickness (BT) of 0.54 on the right second primary molars and of 0.53 mm on the left one, an increase in the buccal alveolar bone height (BH) of 0.72 mm on the right one and of 0.33 mm on the left. Albeit statistically significant, these values were deemed not clinically significant as they were below 1 mm.

The relatively small vestibular movement of the upper first molars, and its direct consequence of buccal tipping and alveolar bone loss, is a direct effect of palatal expansion [37,38]. For periodontal reasons, the deciduous molar should preferably be chosen as anchor teeth because when the permanent second premolars erupt, they reduce the periodontal side effects of maxillary expansion [39]. 

The increase in buccal bone height (BH) and the reduction of buccal bone thickness (BT) at the level of 6-6 did not appear statistically significant and this validates the control and clinical safety of this low compliance Ni–Ti palatal expander. Any variation in the vertical alveolar bone directly influences BH values as they are taken close to the occlusal border of the bone crest. BT instead is measured in an apical area that is less likely to experience major changes due to tooth displacement after maxillary expansion. The significant decrease in BH for E-E is allegedly due to the greater stress they are suffering as anchorage teeth, resulting in greater bodily movement compared to the first molars. 

Mixed results have been reported in the literature, this event is largely attributable to the different methodologies used (CBCTs, experimental settings, measurement protocol). 

Few articles have discussed the alveolar bone thickness related to maxillary expansion procedures [18,40]. Our study is the first that quantitatively assessed alveolar bone measurements by means of CBCT in patients treated with a Ni–Ti leaf spring expander. Weissheimer observed [41] a reduction in BT of 0.5 mm. Brunetto et al. [24] recently investigated the effect of two protocols of screw activation (rapid and slow) with a Haas palatal expander on the BT of the first molars. Brunetto et al. highlighted that both rapid and slow palatal expanders produce significant vestibular displacement of the maxillary first permanent molars. Reduction in BT and BH were found in both groups, and particularly in patients that underwent slow maxillary expansion. In fact, dental and periodontal structures could be influenced by the frequency of the activation of the maxillary appliance device. 

When compared, our results were different to those published by Brunetto et al. [24]. A bigger detriment on the alveolar BT and BH was found on the first permanent molar in their study with both slow and rapid activations of the screw. This difference could be due to the fact that in our study, the maxillary expander was anchored on the second deciduous molar, thus protecting the periodontal structures of the first permanent molar as mentioned by Digregorio et al. [42].

Digregorio et al. demonstrated that a rapid maxillary expander that used the second primary molars as anchor teeth did not affect the BT on the upper permanent first molars. In contrast, if second primary molars were not available as anchor teeth and primary molars were to be banded for anchorage, the appliance caused a reduction in BT [42].

As reported by Baccetti et al. [43,44], the period before puberty is the most indicated stage to treat a transverse maxillary deficiency. In fact, patients undergoing RME during this specific stage of development showed greater and more stable skeletal changes in all involved maxillary structures. As the patients considered in the present study were treated before the pubertal peak of growth, further research with Ni–Ti leaf spring palatal expander during or after the peak of growth is required.

Another limitation of the current study was the difficulty in obtaining multiple CBCT of a subject to allow a better understanding of the alveolar bone thickness and height longitudinal changes over time. The continuous development of three-dimensional radiation-free techniques like magnetic resonance imaging (MRI) will hopefully help with this particular problem in the future [24,45,46,47,48]. Another main limitation of the present study was a relatively small sample (albeit sufficient for inferential statistics consideration), so the results of our study should be taken with caution.

Further research on the periodontal effects of maxillary expansion should be done with a greater sample size, especially with a RME control group, which could be useful to better understand the differences with the aforementioned described technique. 

## 5. Conclusions

The present research seems to sustain the absence of a statistically significant bone remodeling vestibular to the first permanent molars when maxillary expansion is carried out on deciduous second molars with a Ni–Ti spring maxillary expander. Therefore, the use of a maxillary expander with Ni–Ti leaf springs appears to be a safe procedure in the treatment of maxillary hypoplasia during mixed dentition.

## Figures and Tables

**Table 1:** Descriptive statistics with mean, standard deviation (SD), confident interval, I–III interquartile and the Student’ paired t-test between T1 and T2 for the Leaf Expander treatment.

Variable	Timing	Mean ± SD	Confidence Interval	I–III Interquartile	Shapiro Wilk	Pairwise Comparison *
BT-E-r (mm)	1	1.31 ± 0.47	(1.06–1.56)	(0.78–1.80)	0.056	<0.001 *
BT-E-r (mm)	2	0.77 ± 0.36	(0.58–0.97)	(0.56–0.98)	0.008	
BT-E-l (mm)	1	1.35 ± 0.43	(1.12–1.59)	(1.08–1.74)	0.366	0.001 *
BT-E-l (mm)	2	0.88 ± 0.36	(0.69–1.07)	(0.60–1.02)	0.095	
BT-6-r (mm)	1	2.41 ± 0.39	(2.20–2.62)	(2.10–2.80)	0.193	0.107
BT-6-r (mm)	2	2.18 ± 0.38	(2.25–2.66)	(1.97–2.49)	0.682	
BT-6-l (mm)	1	2.14 ± 0.45	(1.90–2.38)	(2.13–2.84)	0.665	0.074
BT-6-l (mm)	2	2.07 ± 0.38	(1.87–2.28)	(1.84–2.50)	0.391	
BH-E-r (mm)	1	7.00 ± 0.65	(6.65–7.35)	(6.42–7.42)	0.109	0.008 *
BH-E-r (mm)	2	7.72 ± 0.80	(7.30–8.14)	(6.99–8.53)	0.113	
BH-E-l (mm)	1	6.80 ± 0.58	(6.49–7.10)	(6.38–7.19)	0.562	0.020 *
BH-E-l (mm)	2	7.13 ± 1.70	(6.22–8.04)	(6.92–7.92)	0.000	
BH-6-r (mm)	1	7.55 ± 0.71	(7.17–7.93)	(7.11–8.10)	0.568	0.181
BH-6-r (mm)	2	8.17 ± 0.78	(7.76–8.58)	(7.48–8.87)	0.422	
BH-6-l (mm)	1	7.60 ± 0.65	(7,25-7,95)	(7.14-7,94)	0.401	0.118
BH-6-l (mm)	2	8.06 ± 0.91	(7.56–8.74)	(7.10–8.73)	0.193	
TIP E (°)	1	52.78 ± 10.11	(47.39–58.16)	(44.75–61.53)	0.176	0.067
TIP E (°)	2	60.20 ± 11.36	(54.14–66.24)	(50.16–66.11)	0.364	
TIP-6 (°)	1	57.17 ± 7.03	(53.42–60.91)	(50.42–62.60)	0.501	0.539
TIP-6 (°)	2	55.72 ± 7.87	(51.53–59.91)	(48.76–60.37)	0.796	
IW-E (mm)	1	29.5 ± 0.26	(2.81–3.09)	(2.78–3.079)	0.657	<0.01 *
IW-E (mm)	2	34.3 ± 0.23	(3.31–3.56)	(3.27–3.619)	0.930	
IW-6 (mm)	1	30.5 ± 0.32	(2.88–3.22)	(2.85–3.24)	0.099	0.002 *
IW-6 (mm)	2	33.2 ± 0.27	(3.17–3.46)	(3.06–3.46)	0.637	

Statistical significance was set at *p* < 0.05; * *p* < 0.05.
